# 4'-Hydroxywogonin suppresses lipopolysaccharide-induced inflammatory responses in RAW 264.7 macrophages and acute lung injury mice

**DOI:** 10.1371/journal.pone.0181191

**Published:** 2017-08-08

**Authors:** Chao Fan, Le-Hao Wu, Gu-Fang Zhang, Fangfang Xu, Shuo Zhang, Xiuli Zhang, Lei Sun, Yang Yu, Yan Zhang, Richard D. Ye

**Affiliations:** 1 School of Pharmacy, Shanghai Jiao Tong University, Shanghai, China; 2 Dalian Institute of Chemical Physics, Chinese Academy of Sciences, Dalian, China; 3 Institute of Chinese Medical Sciences, University of Macau, Macau Special Administrative Region, China; National Institutes of Health, UNITED STATES

## Abstract

4'-Hydroxywogonin (4'-HW), a flavonoid, has been isolated from various plants and shown to inhibit NO production in macrophages. However, the molecular mechanisms and its in vivo activity have not been determined. Our study aimed to investigate the mechanisms underlying the anti-inflammatory effects of 4'-HW in vitro and in vivo. We showed that 4'-HW potently reduced the expression levels of COX-2 and iNOS as well as their products, prostaglandin E_2_ (PGE_2_) and nitric oxide (NO) respectively, in LPS-stimulated RAW 264.7 macrophages. 4'-HW also suppressed LPS-induced pro-inflammatory cytokines at mRNA and protein levels. Moreover, 4'-HW blocked the interaction of TAK1 and TAB1 in LPS-stimulated RAW 264.7 macrophages, resulting in an inhibition of the TAK1/IKK/NF-κB signaling pathway. Furthermore, 4'-HW also reduced the phosphorylation of MAPKs and PI3/Akt signaling pathways in LPS-stimulated RAW 264.7 macrophages. 4'-HW was also significantly decreased the intracellular reactive oxygen species (ROS) level. The effect of 4'-HW was confirmed in vivo. 4'-HW exhibited potent protective effect against LPS-induced ALI in mice. These findings indicate that 4'-HW suppresses the LPS-induced response in vitro and in vivo. It is likely that the inhibition of the TAK1/IKK/NF-κB, MAPKs and PI3/AKT signaling pathways contribute to the anti-inflammatory effects of 4'-HW. Our study suggests that 4'-HW may be an important functional constituent in the plants and has the potential value to be developed as a novel anti-inflammatory agent.

## 1. Introduction

Inflammation is an adaptive response triggered by noxious stimuli and conditions, such as infection and tissue injury [1]. A wide range of progressive diseases, including cancer, neurological disease, metabolic disorders and ALI, are associated with inflammation [2, 3]. Inflammation is regulated by an array of mediators including cytokines. Macrophages, as the main pro-inflammatory cells, release various inflammatory cytokines such as tumor necrosis factor-α (TNF-α), interleukin-6 (IL-6) and interleukin-1β (IL-1β), and other inflammatory mediators, such as nitric oxide (NO) and prostaglandin E_2_ (PGE_2_) upon activation by bacterial products such as LPS [4]. Suppressing the expression of these pro-inflammatory mediators and cytokines, therefore, could ameliorate the inflammatory diseases [5].

LPS, the major constituent of the outer membrane of Gram-negative bacteria, initiates a signaling cascade through its interaction with Toll-like receptor 4 (TLR4) [6]. Once TLR4 is activated by LPS, a complex of proteins, such as TNF receptor-associated factor 6 (TRAF6), are recruited. Subsequent association of TRAF6 and TAK1 activates the downstream signaling molecules NF-κB and MAPKs [7]. NF-κB is a homo- or heterodimer consisting of five different transcription factor proteins: (RelA), c-Rel, Rel-B, p50 and p52. The NF-κB dimers exist in an inactive form in the cytoplasm and bound to the inhibitory protein of NF-κB (IκB), of which the prototypical member is IκB-α. Upon pro-inflammatory signal stimulation, IκB-α becomes phosphorylated by IκB kinase (IKK) [8]. After IκB has been phosphorylated, it is ubiquitinated and degraded, resulting in the translocation of released NF-κB to nucleus where it binds to κB-binding sites in the promoter regions of target genes and induces the transcription of pro-inflammatory mediators [9]. The MAPKs including extracellular signal regulated kinase (ERK), c-jun N-terminal kinase (JNK) and p38, regulate a broad range of cellular events including gene expression, mitosis, differentiation and apoptosis [10, 11]. The MAPK families also mediate inflammatory and immune responses, and their signaling pathways are involved in LPS-induced iNOS expression in macrophages [12]. Moreover, several studies have shown that MAPKs play critical roles for the activation of NF-κB [13]. PI3K/AKT signaling can be activated by LPS-induced TLR4-mediated pathway upstream of NF-κB, and play a critical role in NF-κB activation in inflammatory responses [14]. Therefore, substances that could inhibit LPS-induced signaling pathway could also alleviate the development and procession of inflammatory diseases.

**Fig 1:**
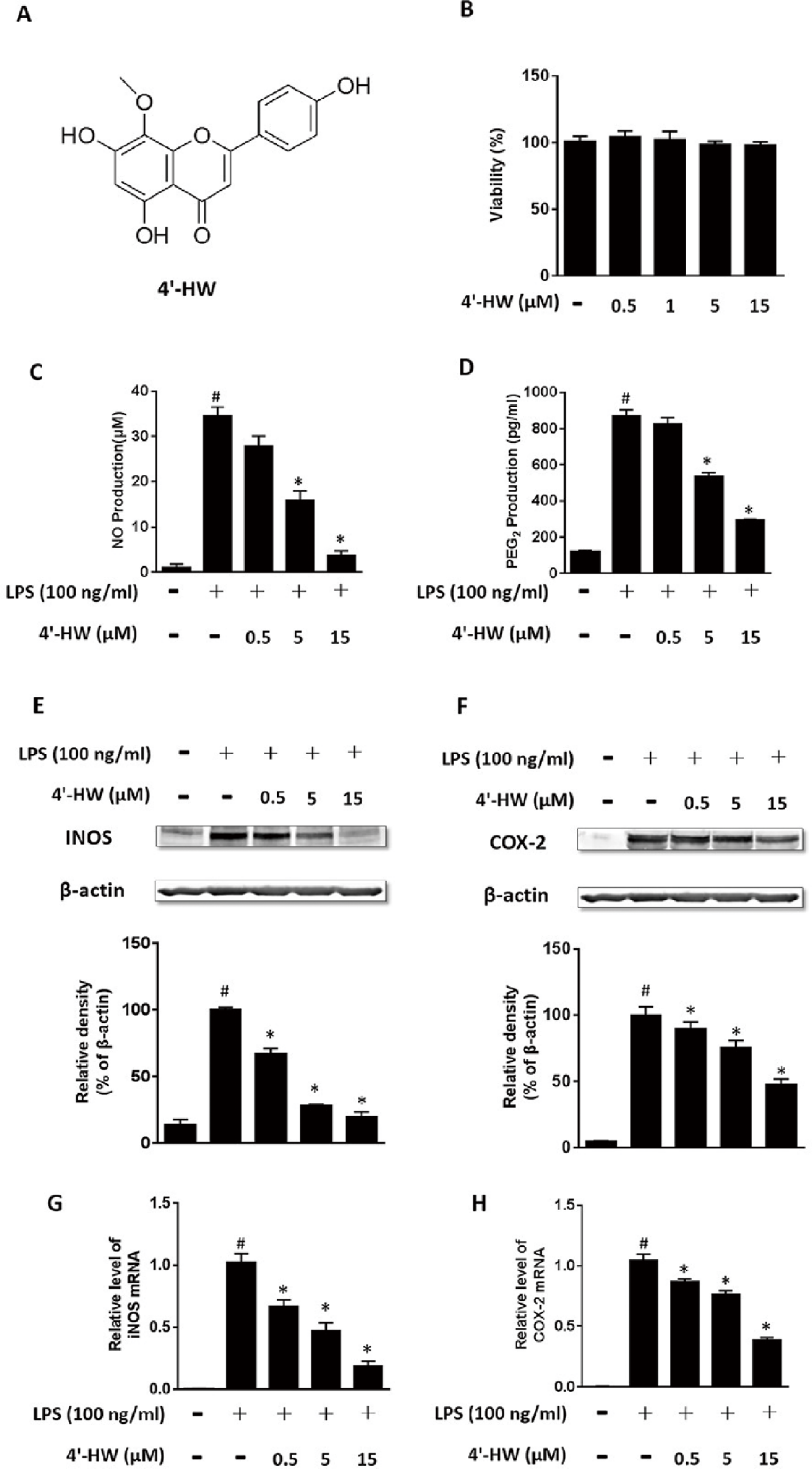
4'-HW abrogated LPS-induced production level of NO and PGE2 through inhibition of iNOS and COX-2 expression in RAW 264.7 macrophages. (A) The chemical structure of 4'-HW. (B) Cytotoxicity in RAW 264.7 macrophages treated with 4'-HW (0.5, 1.5 and 15 μM) for 24 h. (C, D, E, F) Cells were treated with 0.5, 5 and 15 μM 4'-HW for 1h prior to the addition of LPS (100 ng/mL) for an 24 h incubation. NO and PGE2 levels were determined with Griess regent and a commercial kit, respectively. The protein level of iNOS and COX-2 was determined by Western blot analysis using specific antibodies. The immune-reactive bands were quantified using the NIH Image J software. (G and H) Cells were pretreated with 4'-HW for 1 h prior to the addition of LPS (100 ng/mL), and then cells were further incubated for 4 h. The mRNA levels of iNOS and COX-2 were determined by quantitative real-time PCR. The data shown represent the mean ± SD of three independent experiments. #p<0.05 vs the control group; *p<0.05 vs the LPS-treated group.

4'-HW (Structure seen Fig 1A) has been isolated from a variety of plants including *Scutellaria barbata* and *Verbena littoralis* [15, 16]. It has been reported to inhibit LPS-induced NO production in RAW 264.7 macrophages [17], demonstrating anti-inflammatory potentials. However, the molecular mechanisms are not elucidated and the in vivo anti-inflammatory activity are not determined. This study aimed to investigate the mechanisms underlying the anti-inflammatory effects of 4'-HW and its protective effect against LPS-induced ALI in a murine model.

## 2. Materials and methods

### 2.1. Materials

4'-HW (purity >98%) used for this study was purchased from Chem Faces (Wuhan, China). Dulbecco’s modified Eagle’s medium (DMEM), fetal bovine serum (FBS), penicillin, and streptomycin were purchased from Life Technologies Inc. (Grand Island, NY, USA). LPS (Escherichia coli, serotype 055:B5), 3-(4, 5-dimethylthiazol-2-yl)-2, 5-diphenyltetrazolium bromide (MTT), and Dimethyl sulfoxide (DMSO) were purchased from Sigma Chemical Co. (St. Louis, MO, USA). Antibodies against iNOS, COX-2, p-JNK, JNK, p-ERK, ERK, p-p38, p38, IκBα, IKKα, IKKβ, p-IKKα/β, p-p65, p65, p-AKT, AKT and β-actin were purchased from Cell Signaling (Boston, MA, USA).

### 2.2. Animals

Male C57BL/6 mice, 6–8 weeks old and weighing approximately 20 g, were purchased from SLACCAS Laboratory Animal Co., Ltd (Shanghai, China). All mice were housed (four to five animals per cage) with a 12/12 h light/dark cycle, with ad libitum access to food and water. The housing, breeding, and animal experiments were in accordance with the National Institutes of Health Guide for the Care and Use of Laboratory Animals, with procedures approved by the Biological Research Ethics Committee of Shanghai Jiao Tong University.

### 2.3. Cell culture

The RAW 264.7 macrophage cell line was obtained from the China Cell Line Bank (Beijing, China). The cells were cultured in DMEM medium supplemented with 10% FBS, penicillin (100 U/ml), and streptomycin (100 μg/ml) in a 37°C and 5% CO2 incubator, and maintained up to 5 passage.

### 2.4. MTT assay

Cell viability was determined by an MTT assay following the manufacturer’s instructions. Cells were plated overnight in 96-well plates (5×10^3^/well). The cells were treated with 0.1% DMSO (vehicle control) or 4'-HW at various concentrations for 24 h. Following another 4 h incubation with 20 μl MTT solution (5 mg/ml), the culture supernatant was discarded and 100 μl DMSO was added to each well to dissolve the formazan crystal. OD absorbance was recorded at 570 nm with a microplate reader (FlexStation 3; Molecular Devices, Silicon Valley, CA, USA).

### 2.5. Determination of NO production

RAW 264.7 macrophages were plated in 48-well plate (1.5×10^5^/well). After cells became adherent, they were pretreated with different concentrations of 4'-HW for 1 h and then stimulated with 100 ng/ml LPS for an additional 24 h. The nitrite accumulated in culture medium was measured as an indicator of NO production based on Griess reaction. Briefly, 50 μl of cell culture medium was mixed with 50 μl of Griess reagent (1% sulfanilamide in 5% phosphoric acid, 1% α-naphthylamide in H_2_O) in a 96-well plate, incubated at room temperature for 10 min, and then measured at 540 nm using a microplate reader (FlexStation 3, Molecular Devices, Silicon Valley, CA, USA).

### 2.6. ELISA assay

RAW 264.7 macrophages were plated in 6-well plate (1.0×10^6^/well). After cells became adherent, they were pretreated with different concentrations of 4'-HW for 1 h and then stimulated with 100 ng/ml LPS for an additional 24 h. The cell-free supernatants were collected and assayed to determine the levels of TNF-α, IL-6 and IL-1β. In addition, the BALF samples were centrifuged to detect TNF-α, IL-6 and IL-1β levels using an enzyme-linked immunosorbent assay (ELISA) in accordance with the manufacturer’s instruction (eBioscience, San Diego, CA).

### 2.7. PGE_2_ assay

PGE_2_ assay was conducted to detect the concentration of PGE_2_ as previously described [18]. The PGE_2_ levels in macrophage supernatants were done with reagents for a homologous time-resolved fluorescence (HTRF) competitive assay (Cisbio International, France) according to manufacturer’s instructions.

### 2.8. Western blot analysis

Western blot was conducted to detect the protein expression as previously described [19]. Cells were plated in 6-well plates at 2 × 10^6^/well, after cells became adherent, they were pretreated with 4'-HW for 1 h, and then challenged with 100 ng/ml LPS. Cells were then collected and lysed in loading buffer. Nuclear and cytoplasmic proteins were obtained with nuclear and cytoplasmic protein extraction kit (Beyotime Biotechnology, China) according to the manufacturer’s protocol. The cell lysate was analyzed by immunoblot using antibodies against iNOS, COX-2, p-JNK, JNK, p-ERK, ERK, p-p38, p38, IκBα, p-p65, p65, IKKα, IKKβ, p-IKKα/β, p-AKT, AKT and β-actin. Quantification of western blots was performed with Image J software (NIH, Bethesda, MD).

### 2.9. Total RNA extraction and qPCR

Total RNA was extracted from cells using Trizol reagent (Invitrogen, Carlsbad, CA, USA). Reverse transcription of RNA was performed with the Reverse Transcription System A3500 kit (Promega, Madison, WI) according to the manufacturer’s protocol. Relative quantification of gene expression was performed with SYBR® Green Real time PCR Master Mix (TOYOBO, Osaka, Japan) and conducted with the Eppendorf Mastercycler ep realplex (Hauppauge, NY). The following primers were used: TNF-α (5’-TTCTCATTCCTGCTTGTGG-3’; 5’-ACTTGGTGGTTTGCTACG-3’), IL-6 (5’-CTTCTTGGGACTGATG-3’; 5’-CTGGCTTTGTCTTTCT-3’), IL-1β (5’-GATCCACACTCTCCAGCTGCA-3’; 5’-CAACCAACAAGTGATATTCTCCATG-3’), iNOS (5’-GACAAGCTGCATGTGACATC-3’; 5’-GCTGGTA GGTTCCTGTTGTT-3’), COX-2 (5’-TCCAGATCACATTTGATTGA-3’; 5’-TCTTTGACTGTGGGAGGATA-3’). The primers for the mouse housekeeping gene glyceraldehyde-3-phosphate dehydrogenase (GAPDH) were 5’-CCTTCCGTGTTCCTACC-3’ and 5’-CAACCTGGTCCTCAGTGTA-3’.

### 2.10. NF-κB activation

The examination of NF-κB activity was performed by a modification of methods as described previously [20]. THP-1 cells expressing NF-κB-Luciferase were seeded in 96-well plates at a density of 1.5×10^4^ cells per well. On the next morning, the cells were pretreated with 4'-HW or solvent vehicle (0.1% DMSO in culture medium) for 1 h and stimulated with 100 ng/ml LPS for 4 h. Afterwards, the cells were lysed with luciferase lysis buffer and the luminescence of the firefly luciferase were measured in a FlexStation 3 (Molecular Devices,Sunnyvale, CA).

### 2.11. Immunoprecipitation

Cells were treated with 4'-HW (15 μM) with or without LPS (100 ng/ml) for 15 min. Cells were lysed in radioimmunoprecipitation assay (RIPA) buffer and microcentrifuged for 10 min at 14,000 × *g*, 4°C. The supernatant of cell lysate was incubated with primary antibody overnight at 4°C. The immune complexes were allowed to bind to 30 ml of Recombinant Protein G Agarose beads (Invitrogen, USA) at 4°C for 2 h, and the beads were washed three times with lysis buffer. The washed beads were re-suspended in electrophoresis sample buffer and boiled for 10 min. After centrifugation, the supernatants were obtained as immunoprecipitates for Western blot analysis [21].

### 2.12. Immunocytochemistry

Cytoplasmic-nuclear translocation of NF-κB p65 was analyzed by confocal microscopy according to the method described previously [22]. Briefly, RAW 264.7 macrophages were pretreated with or without 4'-HW (15 μM) for 1 h and then treated with LPS (100 ng/ml) for another 30 minutes. Treated cells were fixed and incubated with anti-NF-κB p65 primary antibody, followed by incubation with Alex Fluor®488 donkey anti-rabbit antibody (1:500, Invitrogen) and 5 μg/ml of DAPI, and the fluorescent confocal images were captured using a laser-scanning confocal fluorescence microscope (TCS SP8, Leica Microsystems, Wetzlar, Germany).

### 2.13. Determination of intracellular reactive oxygen species (ROS) production

Intracellular oxidative stress was measured by DCFH oxidation, as described earlier[23]. RAW 264.7 macrophages were plated in 96-well plate (5×10^4^/well). After cells became adherent, they were pretreated with different concentrations of 4'-HW for 1 h and then stimulated with 100 ng/ml LPS for an additional 24 h. The cells were exposed to DCFH-DA for another 1 h and then washed twice with PBS. The fluorescence was measured at 480/530 nm using a microplate reader (FlexStation 3, Molecular Devices, Silicon Valley, CA, USA).

### 2.14. Acute lung injury (ALI) in mice

Mice were randomly divided into five groups (n = 6 per group): control (vehicle), LPS only (3 mg/kg), LPS (3 mg/kg) + 4'-HW (20 or 10 mg/kg), and LPS (3 mg/kg) + dexamethasone (DEX, 5 mg/kg). Mice were intraperitoneally (i.p.) administered twice with vehicle, DEX, two doses (20 and 10 mg/kg) of 4'-HW 12 and 1 h before LPS treatment. Then, LPS was administered Intratracheally to induced lung injury. Mice were killed 6 h after LPS administration under anaesthesia by intraperitoneal injection of sodium pentobarbital to collect bronchoalveolar lavage fluid (BALF), and tissue samples.

### 2.15. Histopathological evaluation

To characterize the histopathological alterations, the collected lung tissues were immersed in 4% fixative for 48 h, embedded in paraffin wax, and cut into 5 μm thick sections. The paraffin-embedded sections were stained with hematoxylin and eosin (H & E) for pathological analysis.

### 2.16. Cell counting and total protein concentration in BALF

The BALF samples were centrifuged to pellet cells. The precipitated cells were re-suspended in PBS to obtain total counts of cell. The protein concentrations of BALF were measured using a BCA protein assay kit in accordance with the manufacturer’s instruction (Nanjing Jiancheng Bioengineering Institute, China).

### 2.17. Measurement of myeloperoxidase (MPO) in lung tissues

To examine the accumulation of neutrophils, the collected lung tissues were homogenized and dissolved in extraction buffer to analyze MPO activity using commercial kits (Nanjing Jiancheng Bioengineering Institute, China) according to the instruction.

### 2.18. Statistical analysis

The results are expressed as mean ± S.E.M. Statistical differences were compared with one-way ANOVA. P values < 0.05 were considered significant. All statistical analyses were carried out using the GraphPad Software (San Diego, CA).

## 3. Results

### 3.1. 4'-HW inhibited NO and PGE_2_ production in LPS-stimulated RAW 264.7 macrophages by suppression of iNOS and COX-2 expression

MTT assay was used to determine the non-cytotoxic doses of 4'-HW in RAW 264.7 macrophages. As shown in Fig 1B, the cell viability was not affected at concentrations up to 15 μM. Using the concentrations of 4'-HW in the range of 0.5–15 μM, NO and PGE_2_ production were measured to elucidate its anti-inflammatory effects in LPS-stimulated RAW 264.7 macrophages. As shown in Fig 1C and 1D, exposure of RAW 264.7 cells to LPS (100 ng/mL) for 24 h markedly increased NO and PGE_2_ production, but the increase was inhibited by 4'-HW dose-dependently. 4'-HW alone did not induce NO and PGE_2_ production in RAW 264.7 cells (data not shown).

4'-HW treatment altered the expression of the respective enzymes for NO and PGE_2_ production. As shown in Fig 1E and 1F, LPS treatment significantly increased the levels of iNOS and COX-2 protein expression in RAW 264.7 cells and these effects were markedly attenuated by 4'-HW in a dose-dependent manner (0.5–15 μM). RT-PCR analysis also showed that 4'-HW (0.5–15 μM) attenuated the increase of iNOS and COX-2 mRNA expression induced by LPS in RAW 264.7 cells (Fig 1G and 1H). These data suggest that 4'-HW can down-regulate LPS-induced iNOS and COX-2 expressions at the transcription level. We noticed that the reduction of COX-2 by 4'-HW is not as impressive as the reduction of iNOS at both protein and mRNA expression level, which was consistent with the inhibition degree of PGE_2_ and NO.

### 3.2. 4'-HW suppressed LPS-induced expression of pro-inflammatory cytokines in RAW 264.7 macrophages

**Fig 2:**
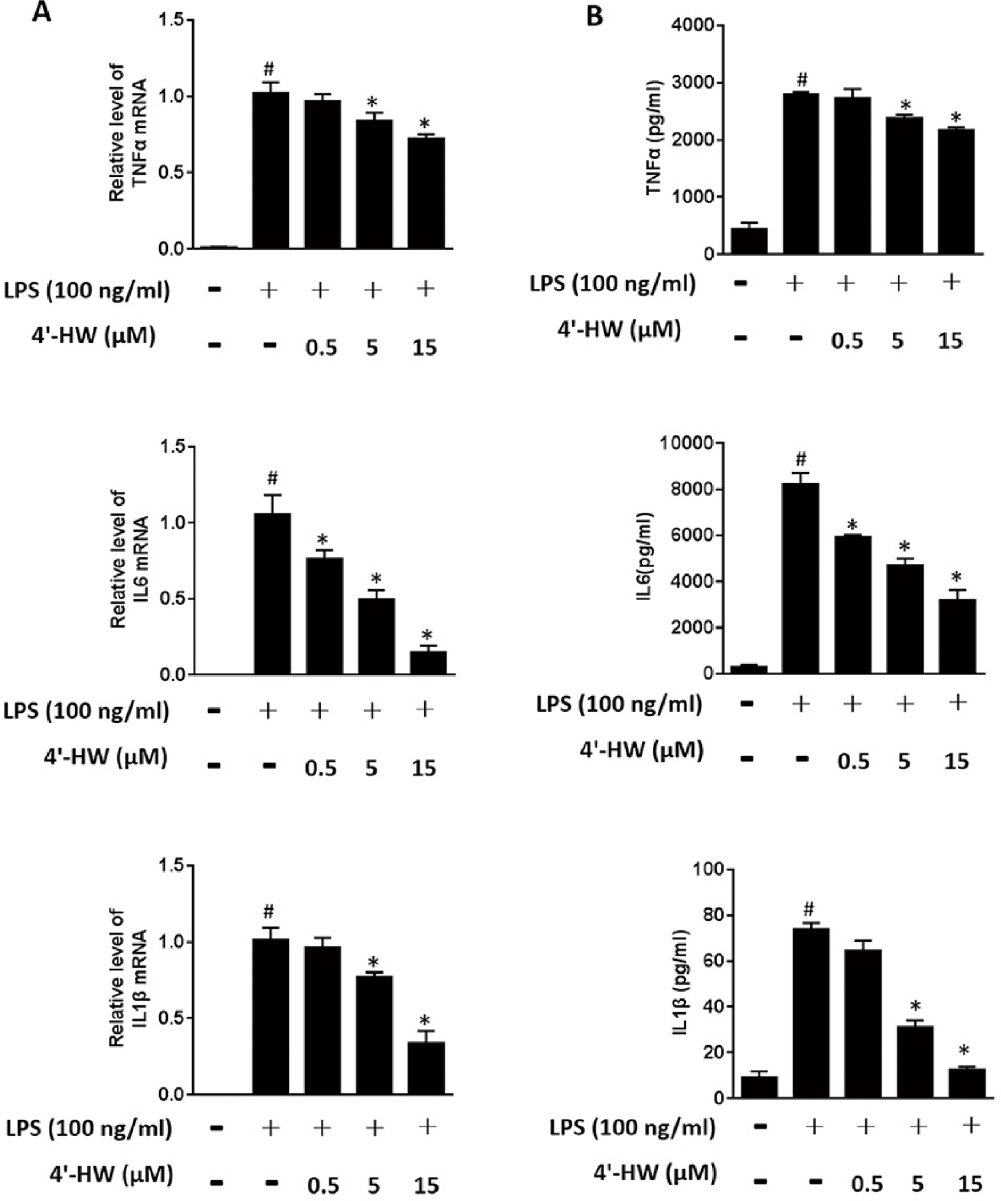
4'-HW suppressed LPS-induced expression of pro-inflammatory cytokines in RAW 264.7 macrophages. (A) The cells were treated with 0.5, 5 and 15 μM 4'-HW for 1 h prior to LPS stimulation (100 ng/mL, 4 h). The transcripts of TNF-α, IL-6 and IL-1β were determined by quantitative real-time PCR. (B) Cells were pretreated with 4'-HW for 1 h prior to stimulation with LPS (100 ng/mL) for 12 h, the Supernatant levels of TNF-α, IL-6 and IL-1β, determined by ELISA. The data shown represent the mean ± SD of three independent experiments. ^#^p<0.05 vs the control group; *p<0.05 vs the LPS-treated group.

Because TNF-α, IL-6 and IL-1β are mainly produced in inflammatory cells, the effects of 4'-HW on LPS-induced production of these pro-inflammatory cytokines in RAW 264.7 macrophages were accessed using RT-PCR and ELISA, respectively. As shown in Fig 2A, exposure of RAW 264.7 cells to LPS (100 ng/mL) for 24 h significantly up-regulated the mRNA expression of TNF-α, IL-6 and IL-1β. Treatment of 4'-HW reduced mRNA expression in a dose-dependent manner, although the reduction effect was noticeably smaller with TNF-α. Similar results were obtained at the protein level when RAW 264.7 cells were treated with 4'-HW before LPS stimulation (Fig 2B).

### 3.3. 4'-HW suppressed LPS-induced activation of NF-κB

**Fig 3:**
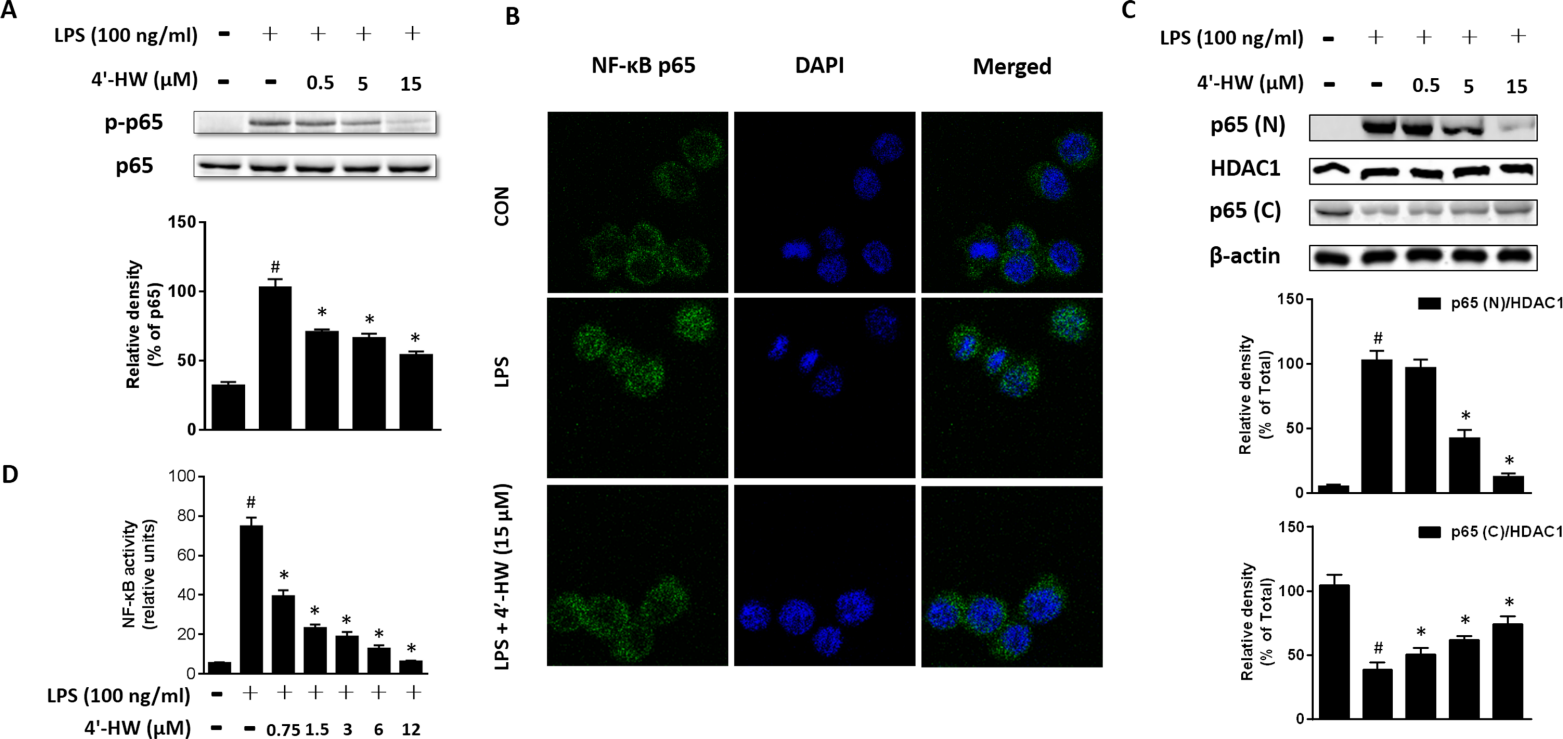
Effect of 4'-HW on LPS-induced NF-κB activation in RAW 264.7 macrophages. (A) Cells were treated with 0.5, 5 and 15 μM 4'-HW for 1 h prior to stimulation with LPS (100 ng/mL, 0.5 h). Cell lysates were analyzed by western blot. (B) Cells were pretreated with 15 μM 4'-HW 1 h and then with LPS (100 ng/mL) for 0.5 h. p65 localization was assessed under a fluorescence microscope as described in *Materials and Methods*. (C) Cells were treated with 0.5, 5 and 15 μM 4'-HW for 1 h prior to stimulation with LPS (100 ng/mL, 0.5 h). Nuclear and cytoplasmic proteins were obtained and analyzed by western blot. (D) THP-1 cells stably transfected with an NF-κB-luciferase reporter were treated with different concentrations of 4'-HW for 1 h prior to LPS stimulation (100 ng/mL, 4 h). The induced luciferase activity was assayed as described under *Materials and Methods*. The data shown represent the mean ± SD of three independent experiments. ^#^p<0.05 vs the control group; *p<0.05 vs the LPS-treated group.

It has been reported that NF-κB is critically required for LPS-induced COX-2 and iNOS activation [24]. Therefore, we examined whether 4'-HW inhibited LPS-induced p65 phosphorylation and nuclear translocation. As shown in Fig 3A, LPS exposure to RAW 264.7 cells for 0.5 h significantly increased the level of phosphorylated p65 subunit. Treatment with 4'-HW markedly attenuated the LPS-induced p65 phosphorylation. Immunofluorescence staining and western blotting analysis confirmed that the LPS-induced nuclear translocation of p65 was reduced by 4'-HW treatment (Fig 3B and 3C).

In subsequent experiment, we tested the effect of 4'-HW on LPS-induced NF-κB activation using THP-1 cells stably transfected with an NF-κB-luciferase reporter. As shown in Fig 3D, 4'-HW potently inhibited LPS-induced expression of the luciferase reporter in a dose-dependent manner. These results indicated that the anti-inflammatory effect of 4'-HW is mediated, at least in part, through inhibition of NF-κB activation.

### 3.4. 4'-HW suppressed LPS-induced degradation of IκB-α and activation of IKK and TAK in RAW 264.7 macrophages

**Fig 4:**
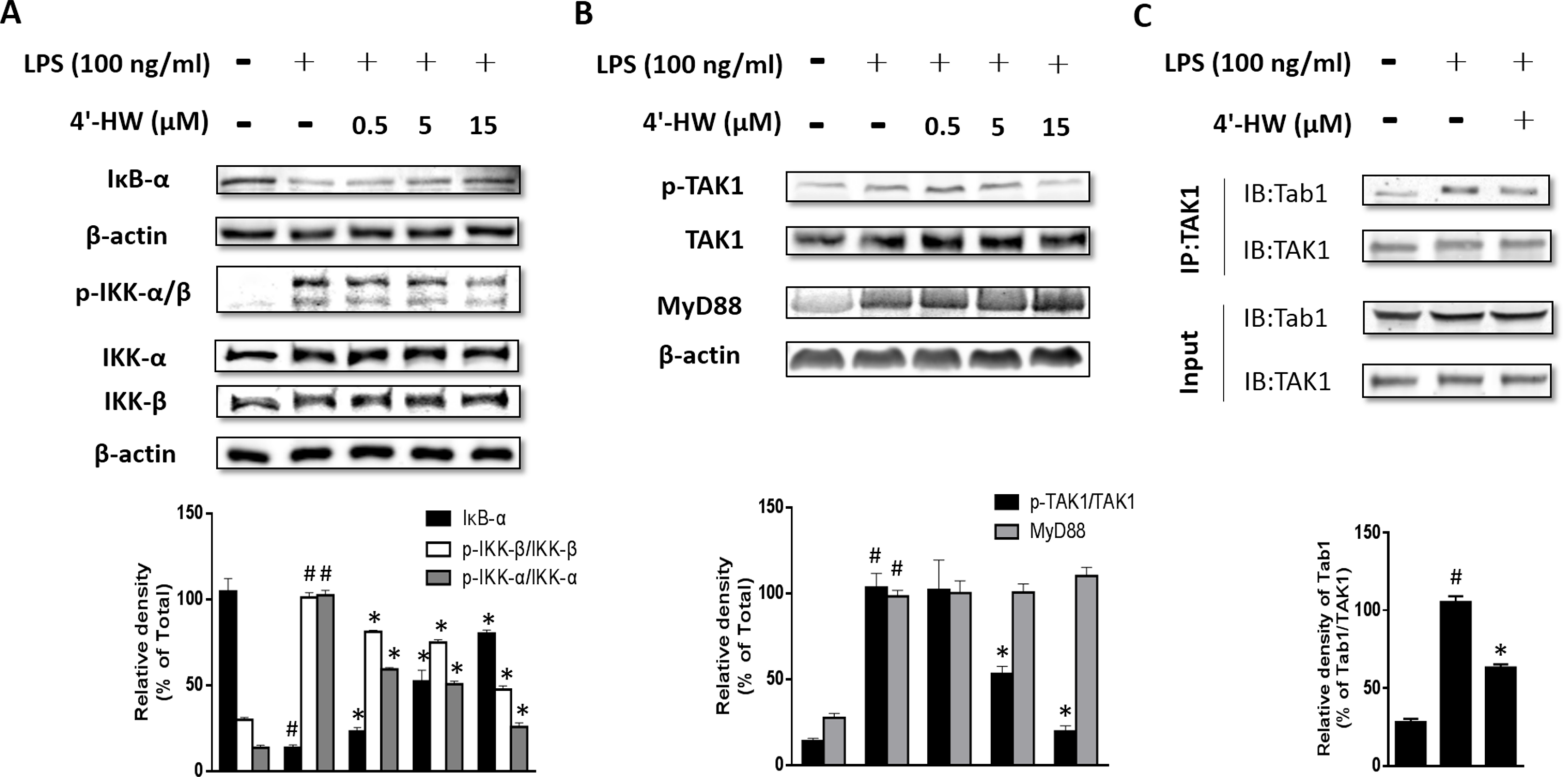
Effects of 4'-HW on LPS-induced expression of IκB-α, IKK-α/β, p-TAK1, MyD88 and the interaction of TAb1 with TAK1 in RAW 264.7 macrophages. (A, B) Cells were treated with 0.5, 5 and 15 μM 4'-HW for 1 h prior to LPS stimulation (100 ng/mL, 15 min). Cell lysates were analyzed by western blot. (C) The effect of 4'-HW on the interaction of TAK1 and TAB1 in LPS stimulated cells. Cells were exposed to 4'-HW (15 μM) for 1 h, then stimulated with LPS (100 ng/mL) for 15 min. Immunoprecipitation assays were conducted to evaluate the binding of endogenous TAK1 and TAB1. The immune-reactive bands were quantified using the NIH Image J software. The data shown represent the mean ± SD of three independent experiments. ^#^p 0.05 vs the control group; *p<0.05 vs the LPS-treated group.

In the classic pathway of activation, NF-κB binds with its inhibitor protein, IκB-α and resides in the cytoplasm in unstimulated cells. Its activation is regulated by the degradation of IκB-α that frees NF-κB and allows it to be translocated to the nucleus [25]. Thus we examined the effects of 4'-HW on LPS-induced degradation of IκB-α. As shown in Fig 4A, LPS induced IκB-α degradation and this effect was attenuated by 4'-HW pretreatment.

Since IKK-α and β are upstream kinases of IκB in the NF-κB signal pathway [26], we examined the effects of 4'-HW on LPS-induced IKK-α/β phosphorylation in RAW 264.7 macrophages by immunoblotting. As shown in Fig 4A, 4'-HW markedly reduced LPS-induced IKK-α/β phosphorylation, whereas 4'-HW did not affect the total amounts of IKK-α and β.

Because TAK1 has been implicated in the regulation of IKK phosphorylation by LPS treatment [27], we further explored whether 4'-HW could suppress LPS-induced phosphorylation of TAK1 in RAW 264.7 macrophages. As shown in Fig 4B, LPS-induced TAK1 phosphorylation was reduced significantly by 4'-HW in a dose-dependent manner, whereas TAK1 protein levels were unaffected regardless of LPS or 4'-HW treatment. To further determine whether 4'-HW affects TAK1 phosphorylation by inhibiting the formation of TAK1/TAB1 complex in the cytoplasm, we performed immunoprecipitation with an anti-TAK1 antibody. Binding of TAK1 to TAB1 was observed after LPS treatment. However, LPS-stimulated RAW 264.7 cells pretreated with 4'-HW showed a reduction in the intensity of the TAK1/TAB1 band (Fig 4C) and the level of immunoprecipitated TAK1 remained unchanged by LPS.

Since the interaction of TLR4 with the adaptor protein MyD88 is critical for TLR4 to activate downstream signaling pathways and induce inflammatory response, we next examined the effect of 4'-HW on the expression of MyD88. As shown in Fig 4B, LPS treatment caused a relative increase in MyD88 expression. To investigate whether 4'-HW could modulate MyD88 expression, the effect of 4'-HW on LPS-induced up-regulation of MyD88 in RAW 264.7 was examined. As shown in Fig 4B, compared to LPS treatment group, the expression of MyD88 was unaffected by 4'-HW treatment.

### 3.5. 4'-HW suppressed the phosphorylation of MAPK and AKT in LPS-stimulated RAW 264.7 macrophages

**Fig 5:**
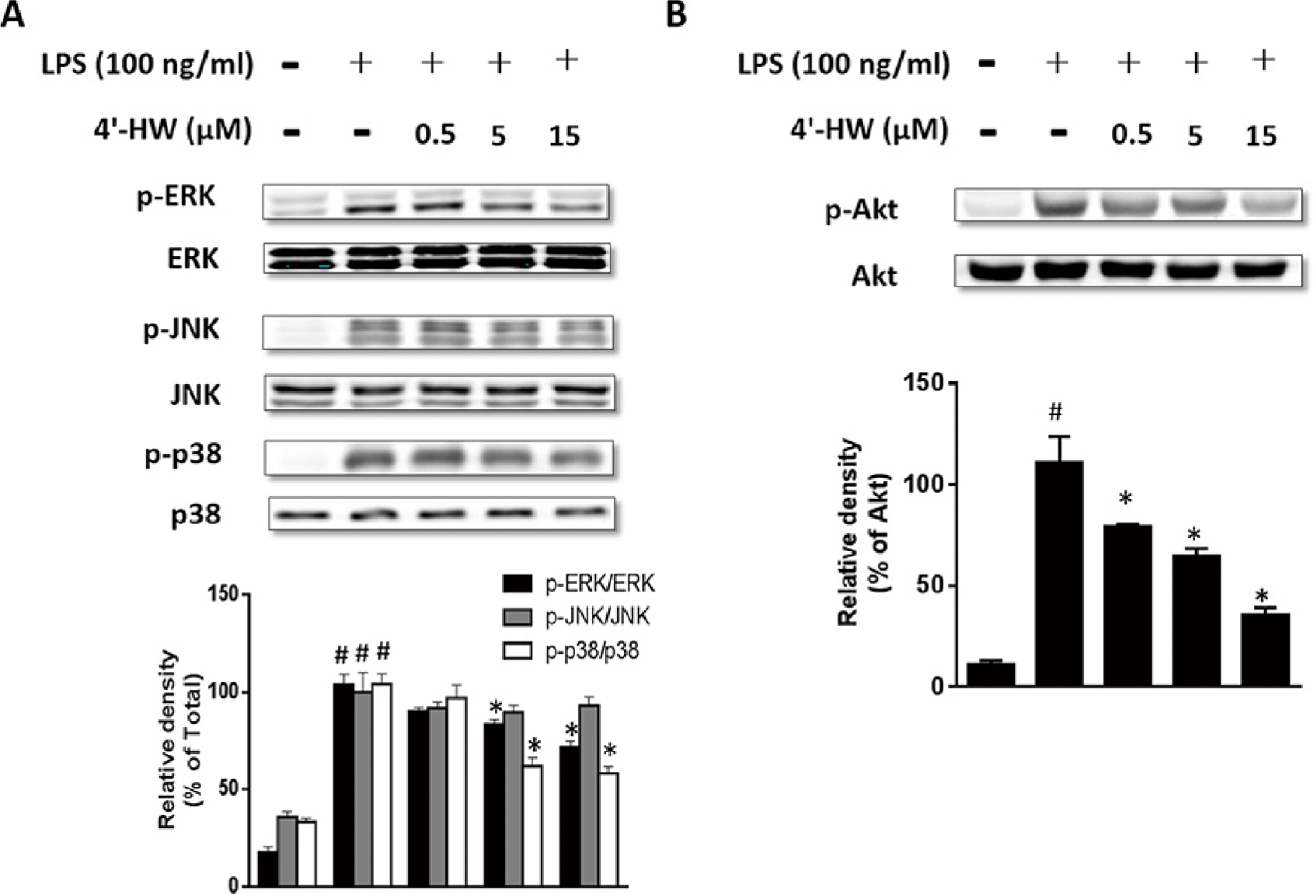
Effect of 4'-HW on LPS-induced activation of (A) MAPKs and (B) AKT in RAW 264.7 macrophages. Cells were treated with 0.5, 5 and 15 μM 4'-HW for 1h prior to LPS stimulation (100ng/mL, 0.5 h). Cell lysates were analyzed by western blot. The immune-reactive bands were quantified using the NIH Image J software. The data shown represent the mean ± SD of three independent experiments. ^#^p<0.05 vs the control group; *p<0.05 vs the LPS-treated group.

To investigate if 4'-HW interferes with LPS-stimulated activation of MAPKs signaling pathways, the phosphorylation of key MAPK signaling proteins were examined by immunoblotting. As shown in Fig 5A, stimulation of RAW 264.7 cells with LPS resulted in an increase in the phosphorylation of ERK1/2, p38 and JNK MAPKs. Pretreatment with 4'-HW for 1 h dose-dependently attenuated the phosphorylation of ERK1/2 and p38 induced by LPS, whereas the phosphorylated JNK was unaffected.

Subsequently, we investigated whether the anti-inflammatory effects of 4'-HW were related to the AKT signaling pathway. As shown in Fig 5B, 4'-HW significantly attenuated LPS-induced phosphorylation of AKT without changing total AKT protein level.

These data suggested that preventing the phosphorylation of ERK1/2, p38 MAPK, and AKT by 4'-HW was attributable for its anti-inflammatory effects.

### 3.6. 4'-HW inhibits ROS production in LPS-stimulated RAW 264.7 macrophages

**Fig 6:**
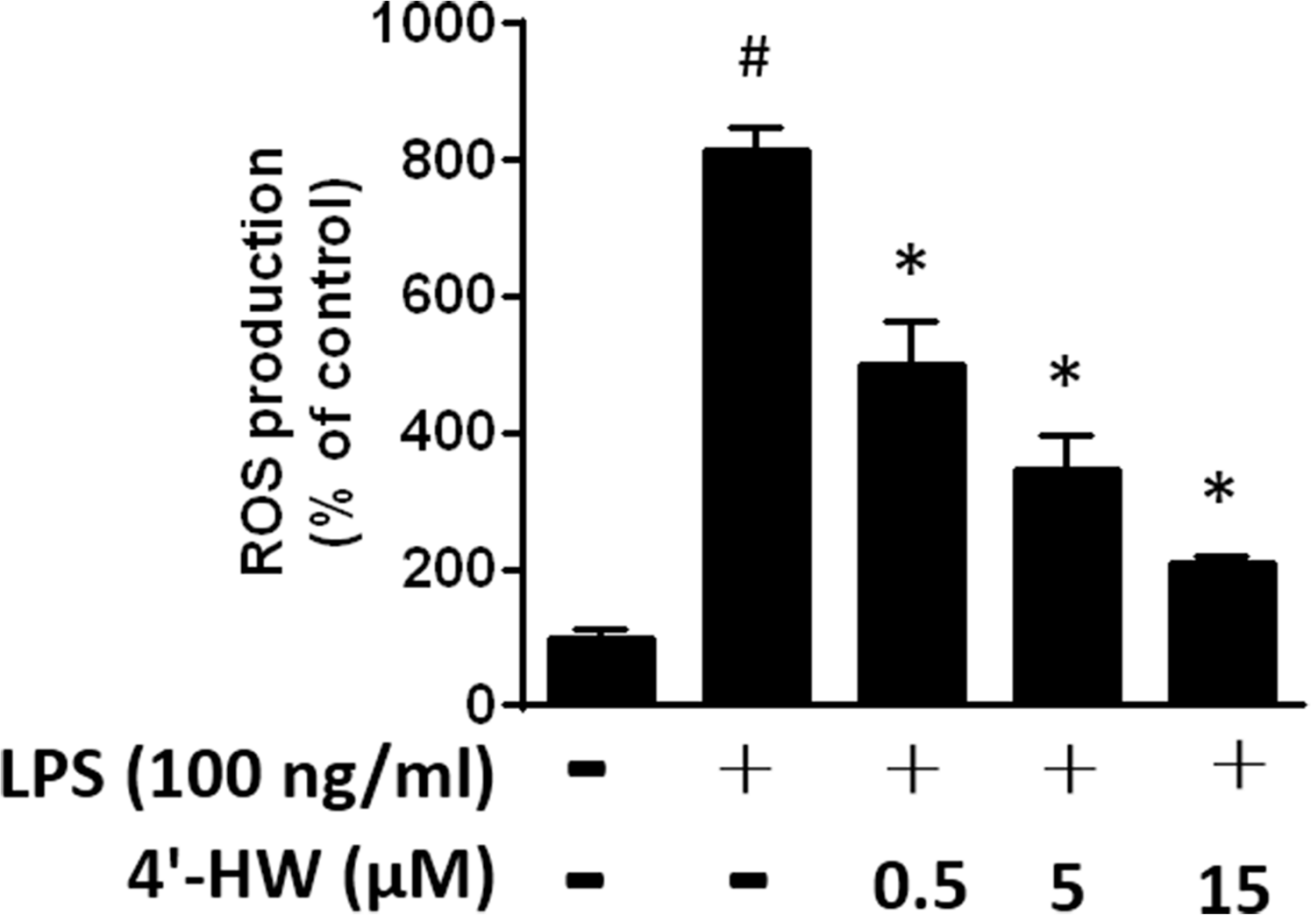
Effects of 4'-HW on LPS-induced ROS production. RAW 264.7 macrophages cells were pretreated with 0.5, 5 and 15 μM 4'-HW for 1 h and then stimulated with 100 ng/mL LPS for an additional 24 h. The ROS production was assayed as described under *Materials and Methods*. The data shown represent the mean ± SD of three independent experiments. ^#^p<0.05 vs the control group; *p<0.05 vs the LPS-treated group.

Previous studies have demonstrated that LPS-induced ROS production is associated with the activation of NF-κB, MAPKs and AKT signaling pathways[28]. Therefore, we examined the anti-oxidant effects of 4'-HW in LPS-stimulated macrophages by measuring the ROS generation. ROS were assessed with the ROS-sensitive fluorophore DCFH. We observed that the level of ROS in response to LPS was significantly higher than that of unstimulated cells, and that treatment of 4'-HW (0.5~15 μM) resulted in a concentration-dependent decrease of measured fluorescence (Fig 6), thus demonstrating its inhibitory effect on ROS production.

### 3.7. 4'-HW alleviated LPS-induced ALI in a mouse model

**Fig 7:**
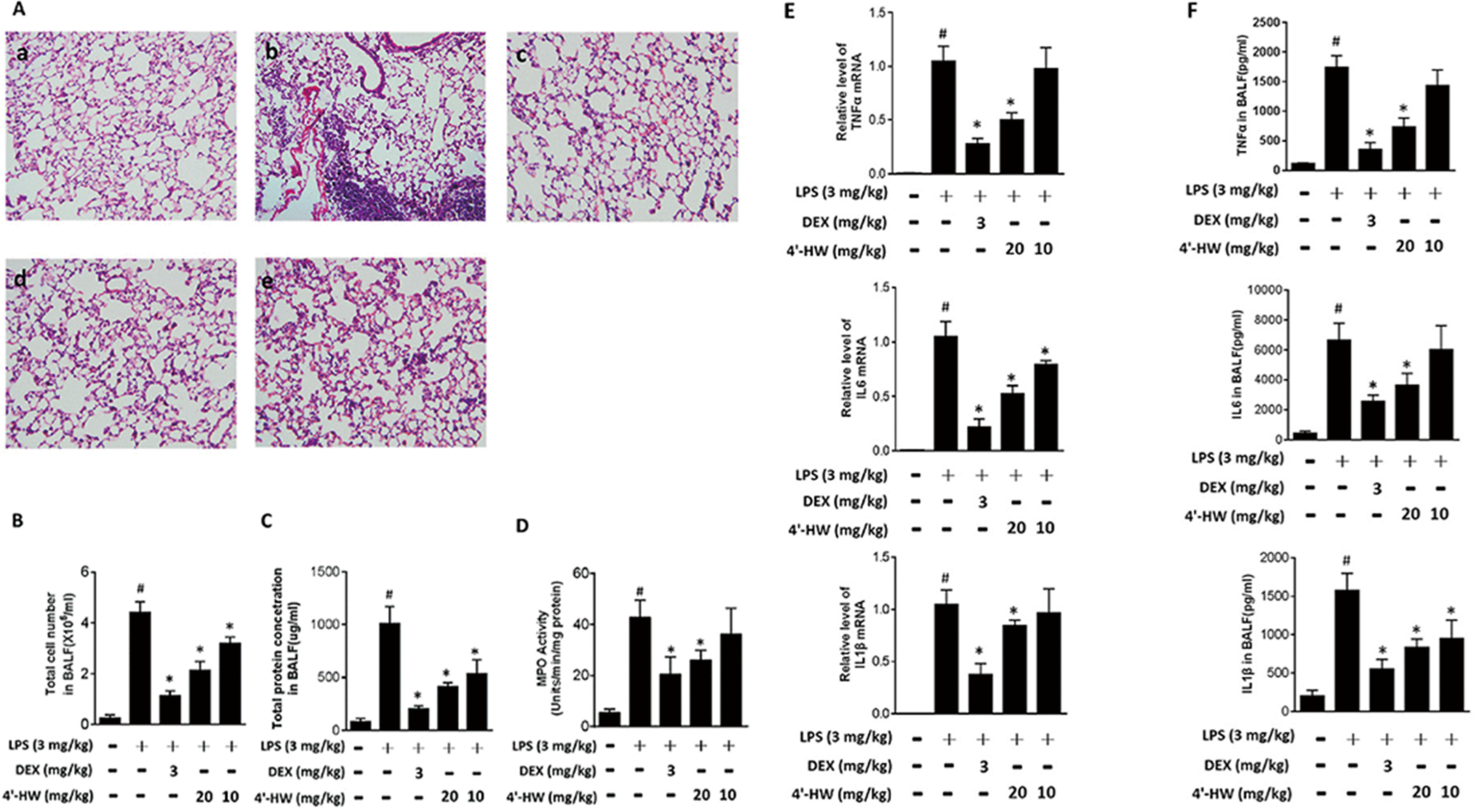
Effect of 4'-HW on LPS-induced acute lung injury in mice. Mice (n = 6 per group) were injected with 4'-HW or vehicle for 12 h and 1 h respectively before LPS injection (3 mg/kg, Intratracheal injection). (A) Lungs from each experimental group were processed for histological evaluation at 6 h after the LPS challenge: (a) control, (b) LPS, (c) LPS + DEX (5 mg/kg), (d) LPS + 4'-HW (20 mg/kg), (e) LPS + 4'-HW (10 mg/kg). (B) The BALF was collected 6 h after LPS challenge to measure the number of total cells. (C) The BALF was collected 6 h after LPS challenge to measure the concentration of total proteins. (D) Effect of 4'-HW on the MPO activity in lung homogenates. (E) the levels of TNF-α, IL-6 and IL-1β in total RNA from lung tissue as determined by quantitative real-time PCR. (F) The BALF levels of TNF-α, IL-6 and IL-1β, determined by ELISA. ^#^p<0.05 vs the control group; *p<0.05 vs the LPS-treated group.

To confirm whether the in vitro anti-inflammatory effects could be validated in vivo, we further examined the potential protective effects of 4'-HW against inflammation in LPS induced ALI mice. Compared to the control group (Fig 7A-a), the lung tissues of mice administered with LPS alone demonstrated marked damage, including a large number of infiltrating leukocytes and lung tissues destruction (Fig 7A-b). Pretreatment with DEX (3 mg/kg) or 4'-HW (20 and 10 mg/kg) significantly attenuated the degree of leukocyte infiltration (Fig 7A-c, 7A-d and Fig 7A-e).

Additionally, as shown in Fig 7B and 7C, the BALF taken from mice exposed to LPS contained more total cells and higher levels of protein concentration compared with the unchallenged groups. Pretreatment with DEX (3 mg/kg) or 4'-HW (10 and 20 mg/kg) significantly decreased the number of total cells and level of protein concentration in BALF, compared to those in the LPS group.

The increase of MPO activity reflects polymorphonuclear neutrophil accumulation in the lung [29]. A further experiment was carried out on MPO activity to evaluate the effects of 4'-HW on LPS-induced ALI. As shown in Fig 7D, LPS greatly increased MPO activity in lung compared to the control group. In contrast, pretreatment with DEX (3 mg/kg) or 4'-HW (10 and 20 mg/kg) significantly reduced MPO activity compared to those in the LPS group without DEX or 4'-HW.

Since pro-inflammatory cytokines play important role in the recruitment of leukocytes into the lungs in LPS-induced ALI, we further determined the levels of cytokines in BALF using qPCR and ELISA assays, respectively [30]. The results were shown in Fig 7E and 7F. Q-PCR results showed that LPS administration markedly increased the expression levels of TNF-α, IL-6 and IL-1β. Pretreatment with DEX (3 mg/kg) or 4'-HW (10 and 20 mg/kg) significantly attenuated the mRNA levels of these three pro-inflammatory cytokines. Similar ELISA results were obtained (Fig 7F). These results suggested that 4'-HW inhibited the level of pro-inflammatory cytokines induced by LPS in BALF.

## 4. Discussion

Inflammation underlies a wide variety of physiological and pathological processes [1]. LPS can potently activate macrophages through TLR4 and induce a variety of pro-inflammatory mediators and cytokines via distinct signaling pathways. Thus, new anti-inflammatory agents are being discovered based on their suppression of pro-inflammatory cytokine and mediator production, and related signal transduction [6]. Our study shows that 4'-HW potently inhibited the expression of COX-2 and iNOS as well as their products, PGE_2_ and NO, in LPS-stimulated RAW264.7. In addition, 4'-HW suppressed LPS-induced pro-inflammatory cytokines at mRNA and protein levels.

We further explored the mechanisms underlying its anti-inflammatory effects. TLR4 signaling through MyD88 leads to downstream activation of TRAF6, resulting in the formation of a complex consisting of TAK1 and TAB proteins, that activates TAK1 by autophosphorylation [31]. Phosphorylation of TAK1 leads to activation of the IKK complex. The IKK complex phosphorylates IκB-α, resulting in its degradation, which allows nuclear translocation of NF-κB and expression of various inflammatory genes [32]. In our study, 4'-HW attenuated LPS-induced TAK1 phosphorylation by inhibiting TAK1-TAB complex formation, IKK phosphorylation, IκB-α degradation and p65 phosphorylation and nucleus translocation, all contributing to the inhibition of the LPS-induced TAK1-IKK-NF-κB signaling pathway. We noticed that 4'-HW didn’t seem to be able to completely abolish the LPS-induced TAK1-TAB1 binding (Fig 4C), which indicated that 4'-HW might not affect TAK1-TAB1 binding directly. Furthermore, 4'-HW also inhibited the phosphorylation of ERK, p38 and AKT. More recent studies have shown that many natural products do regulate NF-κB activity by blocking MAPKs and AKT pathways, including wogonin, that shares a very similar structure with 4'-HW [13, 33–37]. The fact that 4'-HW did not inhibit the expression level of MyD88, while wogonin has been reported to significantly suppressed the LPS-induced expressions of MyD88 [38, 39], suggests that wogonin and 4'-HW may act as different targets. In addition, by comparing the in vitro activities to wogonin, 4'-HW exhibited improved inhibitory effects on NO production (S1 Fig), and mRNA expression of pro-inflammatory cytokines (S2 Fig). Data of the present study indicate that the substitution of 4'-hydroxyl group in ring B of flavone may also contribute to the anti-inflammatory effect of flavonoids in addition to the OHs at C5 and C7 as well as OCH3 at C8 in ring A as reported previously[40]. Further structure-activity relationship remains to be studied. Based on these results, it was presumed that 4'-HW might interfere with the subsequent cell events after LPS binding to TLR4 and the recruitment of MyD88. 4'-HW exerted anti-inflammatory effects, at least in part, by modulating the TAK1-IKK-NF-κB, MAPKs and AKT signaling pathways.

**Fig 8:**
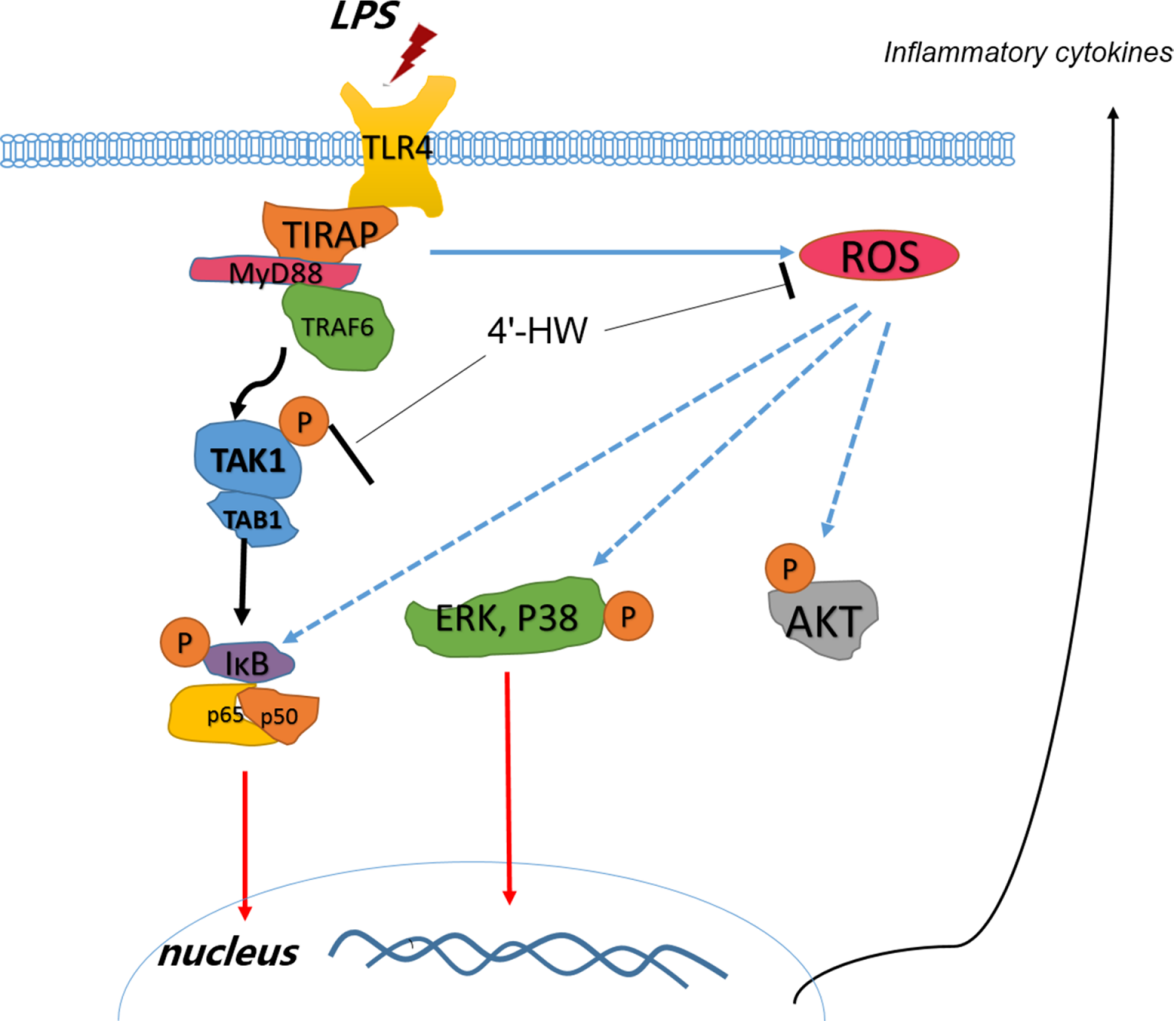
Proposed signaling mechanism for the effects of 4'-HW on LPS-induced inflammation in RAW 264.7 macrophages. 4'-HW exerts its anti-inflammatory activity on RAW 264.7 macrophages by inhibiting ROS production, TAK1/IKK/NF-κB signaling pathway and reducing the phosphorylation of NF-κB, MAPKs and PI3/Akt signaling pathways.

ROS act as signaling molecules triggering various biological responses upon exposure to the stressful environments. Although moderate level of ROS is required for the removal of pathogens and maintenance of cellular hemostasis, overproduction of ROS is thought to be harmful in inflammatory diseases. Thus, modulation of excessive ROS production and oxidative stress would be a promising strategy for the treatment of inflammatory disorders[41]. Moreover, it has been demonstrated that ROS have impacts on several signaling pathways, including NF-κB, MAPKs and AKT[28]. In the present study, LPS-induced ROS production in RAW 264.7 cells was significantly inhibited by 4'-HW. It is likely that 4'-HW may inhibit the activation of NF-κB, MAPKs and AKT by decreasing LPS-induced ROS, which consequently inhibit the kinase activity. The possible mechanism by which 4'-HW may inhibit the anti-inflammatory response is concluded in Fig 8.

Various inflammatory diseases including ALI involves the over-expression of pro-inflammatory cytokines such as TNF-α, IL-6 and IL-1β, and inflammatory mediators such as NO and PGE_2_ via NF-κB, MAPK or AKT pathways in macrophages [42]. In the present study, a murine model of ALI was employed to confirm the in vivo anti-inflammatory activity of 4'-HW. We show that treatment of mice with 4'-HW effectively prevented the LPS-induced lung histopathological changes, neutrophil infiltration, and generation of pro-inflammatory cytokines. In addition, 4'-HW also decreased MPO activity and total cell numbers in the BALF, providing further evidence of a reduction in neutrophil infiltration in the lung [43].

In addition, we have determined in vivo 4'-HW concentration upon a 2-day administration by UHPLC-QTOF-MS. The results showed that detected blood concentration of 4'-HW was low (0.055 μM, 2 h after the second dosing) in ALI mice. However, it is much higher in the lung tissues (0.42 μmol/kg lung). It appears that the pharmacological effects of 4'-HW are produced by the unbound fraction distributed into target tissues after being absorbed into the blood. The representative chromatograms of 4'-HW in serum and lung tissue of ALI mice were shown in S3 Fig.

In summary, the present study demonstrated that 4'-HW exhibited promising anti-inflammatory properties on LPS-induced RAW 264.7 macrophages and protective effect in the model of LPS-induced ALI in mice. These effects were exerted by inhibiting ROS production, thus blocking NF-κB, MAPKs and AKT activation, but not through interfering LPS/TLR4 interaction. Our findings suggest that 4'-HW is a functional constituent in the plants and might be further developed as a novel anti-inflammatory agent.

## Supporting information

S1 Checklist NC3Rs ARRIVE guidelines checklist. (PDF)

S1 Fig The comparison of the effects of 4'-HW and wogonin on LPS-induced production level of NO in RAW 264.7 macrophages. (PDF)

S2 Fig The comparison of the effects of 4'-HW and wogonin on LPS-induced expression of pro-inflammatory cytokines (A) TNF-α, (B) IL-6, and (C) IL-1β in RAW 264.7 macrophages.
(PDF)

S3 Fig Typical chromatograms of 4'-HW in (A) serum and (B) lung tissue of ALI mice.
(PDF)
