# Supplementary material for: 4'-Hydroxywogonin suppresses lipopolysaccharide-induced inflammatory responses in RAW 264.7 macrophages and acute lung injury mice
Source: PLoS One. 2017 Aug 8;12(8):e0181191. doi: 10.1371/journal.pone.0181191 (PMC5549707; doi:10.1371/journal.pone.0181191)
Supplement: S1 Fig — (PDF) [file pone.0181191.s002.pdf]

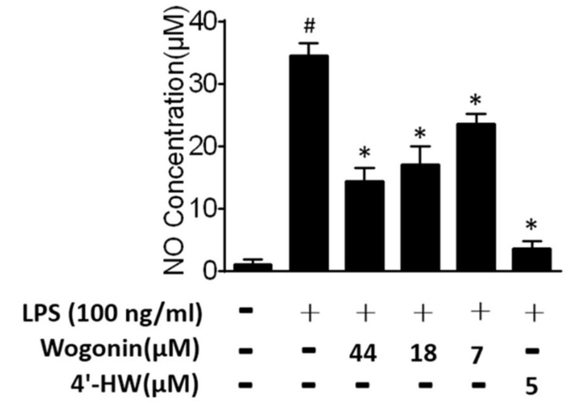

S1 Fig. The comparison of the effects of 4'-HW and wogonin on LPS-induced production level of NO in RAW 264.7 macrophages. Cells were treated with 4'-HW or wogonin for 1h prior to the addition of LPS (100 ng/mL) for an 24 h incubation. NO levels were determined with Griess regent. The data shown represent the mean ± SD of three independent experiments. #p＜0.05 vs the control group; *p＜0.05 vs the LPS-treated group.
