# Supplementary material for: 4'-Hydroxywogonin suppresses lipopolysaccharide-induced inflammatory responses in RAW 264.7 macrophages and acute lung injury mice
Source: PLoS One. 2017 Aug 8;12(8):e0181191. doi: 10.1371/journal.pone.0181191 (PMC5549707; doi:10.1371/journal.pone.0181191)
Supplement: S2 Fig — The comparison of the effects of 4'-HW and wogonin on LPS-induced expression of pro-inflammatory cytokines (A) TNF-α, (B) IL-6, and (C) IL-1β in RAW 264.7 macrophages. (PDF) [file pone.0181191.s003.pdf]

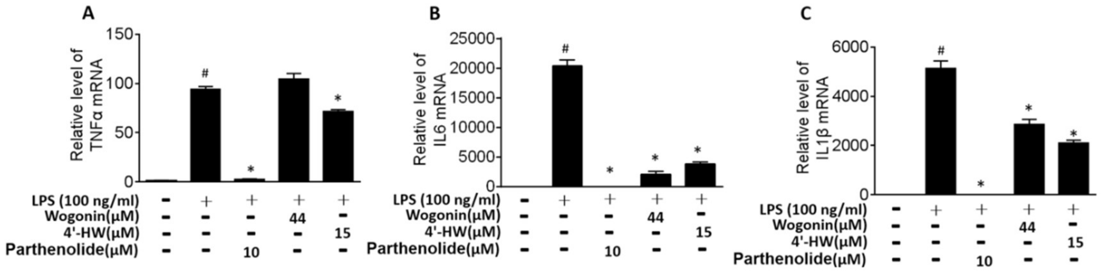

S2 Fig. The comparison of the effects of 4'-HW and wogonin on LPS-induced expression of pro-inflammatory cytokines (A) TNF-α, (B) IL-6, and (C) IL-1β in RAW 264.7 macrophages. Cells were treated with 4'-HW or wogonin or parthenolide for 1 h prior to LPS stimulation (100 ng/mL, 4 h). The transcripts of TNF-α, IL-6 and IL-1β were determined by quantitative real-time PCR. The data shown represent the mean ± SD of three independent experiments. $^{\#}p < 0.05$ vs the control group; $^{*}p < 0.05$ vs the LPS-treated group.
