# Supplementary material for: 4'-Hydroxywogonin suppresses lipopolysaccharide-induced inflammatory responses in RAW 264.7 macrophages and acute lung injury mice
Source: PLoS One. 2017 Aug 8;12(8):e0181191. doi: 10.1371/journal.pone.0181191 (PMC5549707; doi:10.1371/journal.pone.0181191)
Supplement: S3 Fig — Typical chromatograms of 4'-HW in (A) serum and (B) lung tissue of ALI mice. (PDF) [file pone.0181191.s004.pdf]

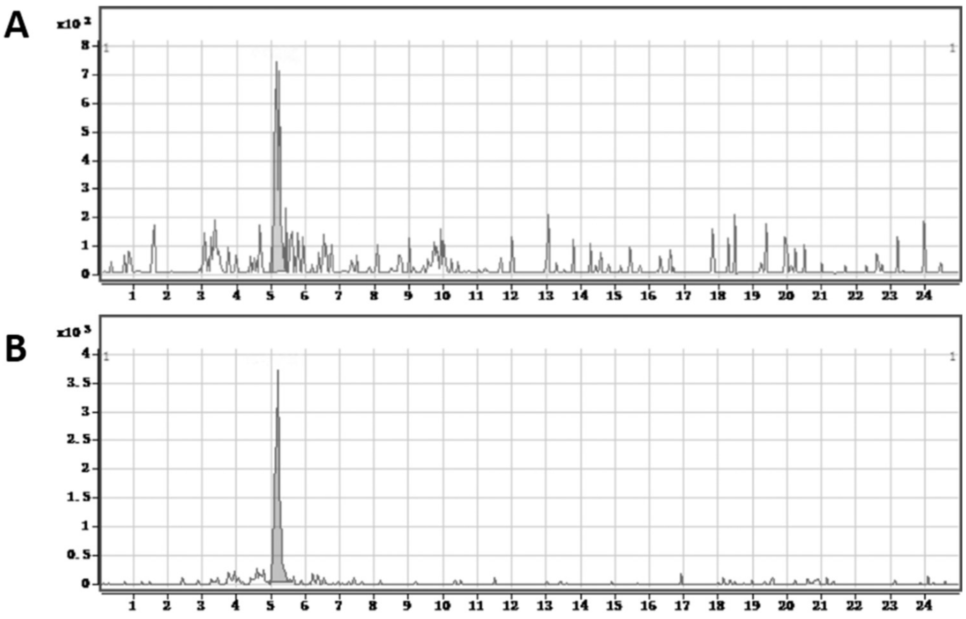

S3 Fig. Typical chromatograms of 4'-HW in (A)serum and (B) lung tissue of ALI mice.

LC-MS analysis

Serum (100 µl) and lung homogenate (150 mg) was extracted with 200 µl and 750 µl methanol, respectively. Following centrifugation and separation, the supernatant was filtered and injected to UHPLC directly. Stock solution of 4'-HW was prepared by dissolving 1.0 mg in 1.0 ml methanol (1 mg/ml). Calibration curves were prepared by diluting the stock solution. The calibration for 4'-HW was constructed by plotting the peak area against the analyte concentrations.

Quantification of 4'-HW was carried out on an ultrahigh-performance liquid chromatograph hyphenated with triple quadrupole tandem mass spectrometer (UHPLC-QTOF-MS) (Agilent 6540, Agilent Technologies, USA). Chromatographic separations of 4'-HW were performed on an Agilent 1290 Infinity UHPC system using a Xterra MS C18 column (2.1 mm x 150 mm, I.D., 5 µm, Waters). A gradient program was used with mobile phase consisting of water (solvent A, containing 0.1% formic acid) and methanol (solvent B) as follows: 0-5 min, 50% B-95% B; 5-15 min, 95% B; 15.1-25 min, 50% B. The flow rate was 0.2 ml/min and the injection volume was 3 µl. The mass spectrometer was equipped with an electrospray ionization (ESI) source. The mass spectrometer conditions were as follows: Nebulizer (N_2) was set at 35 psi. The dry gas was set to 8 L/min. The gas temperature was set to 350 °C. The capillary voltage was set to 3500V. The collision energy was 30 eV. MS scan ranged from100 to 1000 m/z, and the MS/MS scan ranged from 50 to 500 m/z. Spectra were collected in the positive ESI mode. Data was processed using Agilent Masshunter Workstation Qualitative Analysis.
